# Biocrimes, Microbial Forensics, and the Physician

**DOI:** 10.1371/journal.pmed.0020337

**Published:** 2005-09-27

**Authors:** Steven E Schutzer, Bruce Budowle, Ronald M Atlas

## Abstract

Schutzer and colleagues give guidance for physicians who believe that one of their patients has been a victim of an act of bioterror or another biocrime.

Attending physicians, regardless of their specialty or setting of practice, may suspect or learn that their patient has been attacked with a biological agent. In such cases, it is important to be aware of the interactions that may occur with law enforcement, and of the role that the evolving science of microbial forensics [1] may play in the investigation. Physicians are in key positions to preserve critical evidence and, thereby, contribute to the chain of custody, and, at the same time, offer suggestions to help develop the field of microbial forensics.

This article provides guidance to physicians who believe that one of their patients is a victim of an act of bioterrorism or of another biocrime, and who are compelled by law, or with the patient's consent, wish to assist law enforcement in an investigation. In this regard, there are instructive lessons that can be learned from cases of past biocrimes and from analogies to more familiar cases of sexual assault and child abuse (Table 1).

**Table 1:**
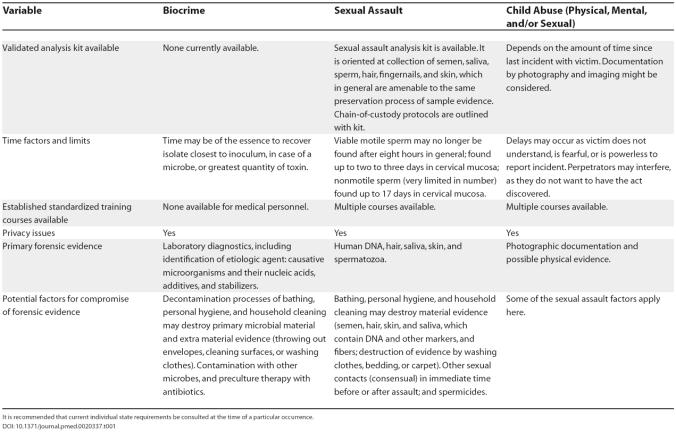
Comparison of Biocrimes, Sexual Assaults, and Child Abuse in the US

It is recommended that current individual state requirements be consulted at the time of a particular occurrence.

## Recent Biocrimes

The most widely publicized bioterrorism event in the United States was the US anthrax mail attacks of 2001. In this case, an astute physician diagnosed the index case of systemic anthrax [2] that set off national panic and a federal investigation that is still ongoing. Highly publicized in Europe was the assassination of a Bulgarian exile in London using ricin, a toxin extracted from castor beans, [3,4] which was delivered to him using an umbrella. Less publicized are other biocrimes such as the case of a laboratory worker in Texas intentionally infecting hospital co-workers with Shigella dysenteriae. Biocrimes are much less likely to occur than many infectious diseases, such as HIV/AIDS. In fact, it is often the abundance of naturally occurring infections that may make the detection of a biocrime difficult. However, there are also documented cases of non-bioterrorism biocrimes (Box 1) [5,6], which far exceed the number of documented bioterrorism acts, as well as many hoaxes where physicians and clinical laboratories were involved in determining if there was a real threat to exposed individuals. [Fig pmed-0020337-g001]

**Box 1. Examples of Non-Bioterror Biocrimes**

- Intentional Salmonella typhi food contamination in France from 1910 to1918
- A Yersinia pestis attack by injection in 1933 in India
- Deliberate use of HIV-infected blood and secretions to inflict harm

Source: [5,6].

**Figure pmed-0020337-g001:**
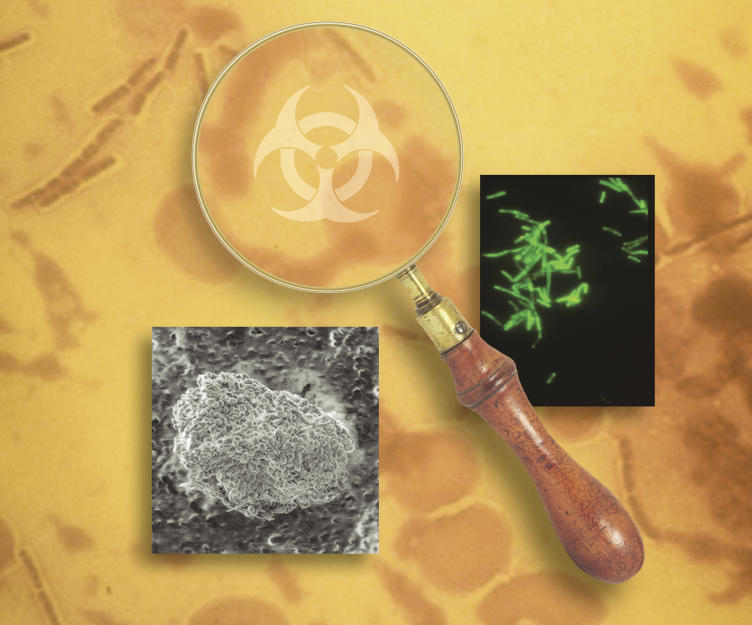
(Reprinted cover image from [30] with permission from Elsevier)

A biocrime is similar to an assault crime, except, instead of a gun or knife, the weapon is a pathogen or a toxin. In the US, acts of bioterrorism are federal crimes that are governed by different responses by law enforcement and public health agencies than those that govern other biocrimes [7]. Most biocrimes and their subset of bioterrorism cases will involve public health agencies because of the nature of a disease threat to the public.

The numerous hoaxes that are biocrimes include white powders found in letters that proclaim the presence of anthrax, and threatening notes claiming ricin contamination of baby food. Ricin currently appears to be a prevalent bioweapon, particularly as a tool for extortion. These potential ricin threats demonstrate the impact of bioterrorism on patient care: physicians had to monitor patients who might have ingested the poisoned food and, hence, were distracted from caring for other patients [8]. Hoaxes can be challenging for the physician, who must distinguish between symptoms and signs that could be toxin-related and those that are just variants of normal health. This challenge was compounded in a recent case where there were trace findings of an inactive form of ricin in baby food. Nonetheless, a hoax is also a crime, and the physician should not discard any evidence simply because material appears innocuous.

As environmental biothreat sensors expand into the workplace and public places, they will be relied on as sentinels for possible biothreat releases. In the US, sensors for anthrax are being placed in some postal offices and at some major public events, and air monitoring is being carried out in major cities and transit systems (the BioWatch system). When such sensors indicate a possible bioterrorist attack, even if the signal is later found to be a false signal, the public will react, and are likely to seek out their own physicians for medical diagnostics, therapy, and advice. Potential cases that follow a public alert warrant evaluation, collection of patient samples, and possibly institution of prophylactic treatments until an alert is deemed a false alarm. Such a situation occurred in March 2005 following a presumptive positive detection of anthrax in a US Department of Defense mailroom. This situation necessitated treating over 900 potential victims with antibiotics as a precaution. However, despite the newer sensor technologies, physicians will likely remain the best and definitive authorities on the presence of an infection.

## Reporting a Biocrime

Patients who believe that they have been a victim of a biocrime generally want both a medical and law enforcement response—that is, they want medical treatment, and they want the perpetrator to be found, prosecuted, and punished. Requirements for how physicians and other members of the health-care system initially report a suspected biocrime are governed at the local level (but regulations are ever-changing, and it is important to check current information at the time it is needed). Many US state regulations mandate that diagnostic laboratories report preliminary isolates of certain microbes to public health authorities, who, in turn, are sometimes compelled to notify law enforcement, depending on the isolate. Certain states have laws that mandate that a physician report specific diseases or unusual clinical manifestations to public health authorities and, in some cases, directly to law enforcement. The latter requirement is analogous to what is expected when there is suspicion of child abuse or gunshot wounds, and failure to notify authorities is itself a crime. The regulations of the Health Insurance Portability and Accountability Act (HIPAA) of 1996 established national standards for health-care transactions as well as security and privacy policy for health-related information, and physicians should be aware of these standards. However, even though these regulations severely restrict the release of patient health–related information, they still permit physicians and health-care facilities to release otherwise protected health information in instances of suspected crimes or threats to public health [9,10].

Given the limited guidance for reporting suspected biocrimes, physicians could face several dilemmas. For example, some patients may not want to report a crime or a disease condition, yet may have reasonable concerns. At the beginning of the HIV/AIDS epidemic, when no treatment was available, a diagnosis of HIV infection caused many patients to fear discrimination and loss of employment, a situation that persists in many parts of the world. Similar questions may arise in bioterrorism events about insurance coverage if an event is deemed an act of war. Communication between physician and patient should help the patient understand the pros and cons of notifying law enforcement of a suspected biocrime, including whether withholding notification could place others at risk. At the very least, discussion can strengthen the doctor–patient relationship.

Fear and embarrassment of reporting potential false alarms to law enforcement or public health authorities may also be a concern for physicians and patients. But if a suspicion is not reported, a critical situation may go unrecognized and continue to worsen. Early notification to law enforcement authorities may provide valuable time and direction for investigative leads. It is expected that there will be many more negatives (false alarms) than positives when alerting law enforcement. Early reports to public health authorities may stem an epidemic. It is a misconception that you must wait for a firm diagnosis before reporting a potential case to authorities. There are many other misconceptions about biocrimes (Table 2).

**Table 2:**
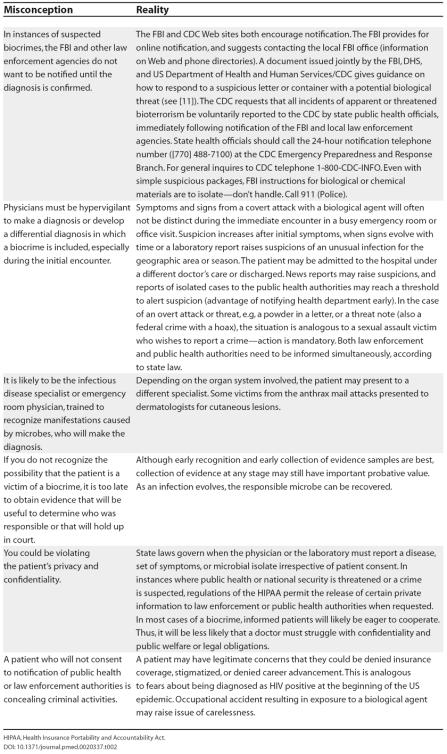
Possible Misconceptions about Biocrimes in the US

HIPAA, Health Insurance Portability and Accountability Act.

Guidance in the US concerning the reporting of suspicions of biocrimes is provided by the Centers for Disease Control and Prevention (CDC; http://www.cdc.gov), the Federal Bureau of Investigation (FBI; http://www.fbi.gov), and the Department of Homeland Security (DHS; http://www.dhs.gov) (Table 2). A joint statement by the FBI, the CDC, and the DHS advises calling the FBI and public health authorities if a suspicious situation arises [11]. Local public health departments are advised to notify the FBI before notifying the CDC. Specifically, “the FBI must be notified for any case of smallpox or pulmonary anthrax, uncommon agent or disease, an illness caused by a microorganism with markedly atypical features, an illness due to aerosol or food or water sabotage, as opposed to a usual transmission route, one or more clusters of illnesses that remain unexplained after a preliminary investigation; deliberate chemical, industrial, radiation or nuclear release” [12]. Calls or online tips should be directed to the FBI (https://tips.fbi.gov). Interpol and the World Health Organization are also developing response plans to help the public and physicians respond to suspected biocrimes and acts of bioterrorism (http://www.who.int/topics/bioterrorism/en).

## The Physician's Role in Collecting Evidence

Although finding the perpetrator of a crime is a law enforcement function, the actions of attending physicians can help with microbial forensics—the scientific discipline dedicated to analyzing evidence from a biocrime or an act of bioterrorism, and that seeks to authenticate a piece of the puzzle for attribution. Implicit in the term attribution is the identification of the responsible party or the exclusion of the innocent [13].

Many physicians are familiar with the treatment of sexual assault victims, and the need to collect and preserve evidence when the patient consents. Sexual assault analysis kits have been validated to preserve semen, saliva, hair, blood, and skin. They also provide instructions on how to maintain a chain of custody to ensure that there has been no tampering with the evidence. Chain of custody is the process that assures integrity of the evidence, and ensures that there is documentation of the time the evidence is handled and each individual handling or examining the evidence. Courses exist in crime scene investigation, evidence collection, and chain of custody of the evidence in suspected sexual assault cases, and there are often well-trained support personnel who can assist patients and physicians. Such evidence collection guidance and support structures are not well-developed for biocrimes.

In contrast to typical human DNA forensic investigations, with microbial forensics, a chain of custody might not be enacted at the initial stage of medical diagnostics. Good diagnostic practices could permit samples to be used as supporting evidence in a criminal investigation. In some cases, samples can be obtained subsequently under a stricter chain-of-custody process. Law enforcement authorities can assist with such documentation processes.

## Microbial Forensics

Microbial forensics includes the full scope of forensic evidence, such as analyses of microbes, materials used to prepare, stabilize, and deliver the toxin or pathogen, and fingerprints, hair, fiber, and pollen [1,14]. The laboratory analyses used for microbial forensics may include molecular sequencing, microbiological cultures, biochemistry, electron microscopy, crystallography, and mass spectrometry. These analyses go well beyond those used for medical diagnoses and epidemiologic investigations [15]. They require, however, the same substances used by the physician for diagnostics, for example, body fluid samples and microbial cultures. In this regard, the physician and the clinical laboratory have critical roles in the collection and initial analyses of samples for microbial forensics.

In the 2001 anthrax letter attacks, the preservation of the initial and subsequent isolates enabled microbial forensic methods to identify the strain in the attacks as the Ames strain of Bacillus anthracis. Analyses were based initially on a method to identify variable-number tandem repeat sequences, and later on, whole genome sequencing. Comparisons with existing strains in culture collections narrowed the likely source to a laboratory as opposed to being obtained directly from nature [16–18]. Fortunately, the initial strain from the Florida patient (the index case) and strains isolated from other victims, as well as spores from the letters, were preserved for future analyses. Microbial forensic analysis is now able, with the help of specialized facilities such as the Institute for Genome Research (TIGR), to determine the whole genome sequence of the approximately 5 million bases of B. anthracis to identify the polymorphisms that may be signatures of the bioweapon [17].

A major thrust of microbial forensics will, therefore, be the analysis of nucleic acids that can relate the genome of the pathogen to specific sources. This analysis is analogous to human DNA forensic analysis, which is being widely used to prosecute criminals and to exonerate the innocent [19]. But there are important differences between the analyses of microbial genomes and those used in human DNA forensics. Because of the sheer number of potential pathogens that could be employed as a weapon, identifying genetic markers for microbes is a more daunting task than identifying human DNA. In the case of human identification, only one species is involved, and it is often possible to identify an individual person. Viruses and most bacteria are haploid. Microbes primarily reproduce asexually, but can also evolve by recombination, horizontal gene transfer, and gene duplication. Therefore, statistical methodologies and interpretation will require different tools than are currently used for comparing and estimating the rarity of (diploid) human DNA profiles [20,21]. Nevertheless, obstacles due to genetic complexity can be reduced by obtaining samples as early as possible.

If physicians suspect a biocrime, they should take steps to ensure the preservation of the diagnostic samples so that they are not prematurely destroyed. Physicians may also advise the patient to preserve additional material that may prove useful for a criminal investigation. Just as in sexual assaults, in a suspected biocrime, the patient's personal articles may carry traditional forensic evidence that is of equal value to the information revealed by the microbe itself. Unlike sexual assault evidence, in a suspected biocrime, procedures used to preserve one particular microbe may be deleterious for other microbes and for physical evidence (such as fingerprints, culture media, isotopes, hair, and environmental material). In addition, procedures useful for preserving one microbe may be insufficient to preserve another that may be unknown at the time.

## History and Physical Examination

The physician's record of the patient's history and physical examination is evidence that can be expected to be part of any public health and forensic investigation (and subsequent legal proceedings) in either a true attack or a hoax. The physician's ability to interpret the clinical history and physical examination may go beyond differential diagnoses—for example, it can help establish timelines of exposure and of the evolution of disease, which will have forensic and public health implications. The physician is positioned to assist in identification and collection of evidence, and initiate the chain of custody that protects the integrity of the evidence (see supporting online material of [1]; [14,22]) or, at a minimum, maintains good medical practice, akin to that used for transfusion of blood products.

## The Case of *Louisiana v. Schmidt*

The case of *Louisiana v. Schmidt*, in which HIV-infected blood was used as the weapon in an attempted murder [23,24], is instructive for the microbial forensics system. A vial of HIV-infected blood was found in the office of a suspect, a gastroenterologist. The challenge for microbial forensics was to provide evidence that this was, or was not, the source of the victim's HIV infection. HIV, an RNA virus, undergoes rapid mutation, so any direct genetic comparison of the donor source and the recipient (the victim) is complicated. In this case, analysis focusing on both rapidly and more slowly mutating genes of HIV proved to be useful. Examination of strains from the vial, the victim, and control samples (known samples from other patients with HIV residing in the same geographic region as the victim) revealed that the viral RNA from the victim was more closely aligned to that from the vial in the suspect's office than to isolates from other patients in the area. The clinical history and clinical laboratory data obtained by attending physicians provided supporting evidence of the uninfected status of the victim prior to this injection. The victim's prior HIV-negative status was documented by blood donation screenings and negative-HIV tests of sexual partners, prior to the injection. The evidence was presented in a US criminal court. Based on the composite epidemiologic and microbial forensic evidence presented, a conviction for attempted murder was obtained.

This case illustrates several points. Sample collection and documentation by the attending physician are paramount to the biocrime investigation. If an attending physician is suspicious about an acquired infection, especially with an organism that is known to mutate rapidly, more frequent sampling and preservation of those samples are important. The sample may contain other clues (the victim, in this case, also was allegedly injected with blood that was hepatitis C positive). These samples could be helpful both epidemiologically, if there were an outbreak, and forensically, if there were an intentional incident. Analysis of these patient samples and other specimens may determine who was the source, and who was the victim. This case showed that even though the earliest isolates were not obtained, when the possibility of a biocrime was considered, there was still sufficient time to obtain valuable specimens, even with this rapidly mutating virus. The case also illustrates that microbial evidence can be informative, but it is rarely the sole deciding evidence. When considered in conjunction with other evidence—in this situation, epidemiological and clinical data—the case was very strong.

Instructive lessons can also be found by reviewing other cases in the literature [5], such as the laboratory technician who poisoned her co-workers with a laboratory stock of Shigella dysenteriae type 2 in muffins [25], and the poisoning of salad bars to skew an election for political gain [26].

Although our primary focus has been on the role that the practicing physician can play, it is important to remember that medical examiners or coroners can also serve as sentinels for discovering acts of bioterrorism and biocrime, as well as collecting pertinent microbial forensic evidence [27]. They have the statutory authority to investigate deaths that are sudden, suspicious, violent, and unattended. Moreover, the medical examiner or coroner may encounter victims that were never examined by practicing physicians. Autopsies can be crucial for diagnosis of unknown infections and for acquiring evidence for subsequent criminal investigations [1,19]. For example, in 1979, in Sverdlovsk, USSR, at least 66 people died during an anthrax outbreak. The official Soviet government position was that the victims were infected by eating contaminated meat. Autopsy data were inconsistent with the proclaimed cause of death, and, instead, supported the proposition that the disease was inhalational anthrax due to an accidental aerosol emission from a secret military weapons facility [28].

Close working relationships should be developed between the medical examiner/coroner and public health and law enforcement entities to alert one another of possible outbreaks (whether natural or intentional) as soon as possible. For suspected attacks, the medical examiner/coroner should immediately collect case-specific death investigation information and establish a chain of custody. Fortunately, medical examiners and coroners have a long-established relationship with law enforcement. However, if there are questions regarding notification or evidence collection, the medical examiner/coroner can contact the proper public health and law enforcement entities (see Table 2).

## Conclusions

Physicians and other health-care providers are positioned to recognize suspicious situations and alert public health and law enforcement officials. This alone may be the most important step physicians can take (Box 2).

**Box 2. Measures That a Physician May Take toward Securing Evidence in Cases of Biocrimes**

- Maintain primary role in caring for the patient, even at the risk of compromising evidence collection.
- Discuss the situation with the patient, including options for interaction with and disclosure to public health and law enforcement officials.
- If permitted by patient consent, or if required by law, alert as early as possible public health authorities and law enforcement, who can provide the necessary expertise or guidance to collect and preserve evidence.
- Do not assume one agency will notify the other in a time-sensitive period. Ensure that notification has occurred.
- Maintain well-documented medical records because documentation of history, physical examination, and patient course may constitute evidence.
- Obtain samples that may serve as evidence early, frequently, and under a defined chain-of-custody process.
- Once a biocrime is suspected, ensure that the clinical laboratory does not discard microbial isolates, but preserves them for forensic analyses or transfers them under a chain-of-custody procedure (along with accessory material such as the transport tube initially used to transport microbial isolates from patient to laboratory). Law enforcement and public health personnel participating in the Laboratory Response Network can provide this assistance.

In cases of biocrimes, physicians may interact with nonmedical authorities—who often do not fully appreciate that a trusting doctor–patient relationship is crucial for proper care and healing, and that information should be private. It is helpful to inform such officials about the importance of the doctor–patient relationship at the outset so they can be sensitive to the obligations of physicians. Just as with a sexual assault case, once there is recognition of the possibility of a bioterrorism act or other biocrime, the physician should discuss the entire situation with the patient, explaining what can be done with the consent of the patient and what actions physicians are mandated to take to comply with public health and legal requirements. This communication will likely strengthen the patient's relationship with the physician. Within the context of microbial forensics, if the patient consents, or the law requires it, the physician can facilitate preservation of evidence. To the extent possible, earlier, more, and serial sampling of evidence is best.

Physicians can ultimately serve their patients by acting, in the traditional role, as a healer, and by working with public health and law enforcement entities to help prevent further attacks and to achieve justice. As with sexual assaults, identification and conviction of the attacker can bring closure and provide a degree of security to the patient, who can then evolve from being a victim to being a survivor [29]. Physicians and their colleagues are likely to have creative ideas to contribute to the field of microbial forensics. Their input is encouraged and welcomed.

## Abbreviations

CDC: Centers for Disease Control and Prevention
DHS: Department of Homeland Security
FBI: Federal Bureau of Investigation
